# Emotion regulation and its association with subjective memory performance among undergraduate students at port said university

**DOI:** 10.1186/s40359-026-05679-9

**Published:** 2026-10-19

**Authors:** Sama Essam Ismail, Ahmed Gamal Sharaf Eldin, Nihal Sharaf Eldin, Nesrine Saad Farrag

**Affiliations:** 1 Faculty of Medicine, Port Said University, Port-Said, Egypt; 2 Community Medicine Department, Faculty of Medicine, MPH, Port Said University, Port-Said, Egypt; 3 Community Medicine Department, Faculty of Medicine, MD, Port Said University, Port-Said, Egypt

**Keywords:** Emotion Regulation, Subjective Memory Performance, Cognitive Reappraisal, Expressive Suppression, Prospective Memory, Retrospective Memory

## Abstract

**Background:**

Emotion regulation (ER), particularly through cognitive reappraisal (CR) and expressive suppression (ES), plays an important role in psychological well-being and higher-order cognitive functioning. Both ER and subjective memory performance (SMP), as measured by the Prospective and Retrospective Memory Questionnaire (PRMQ), have been proposed to draw on shared executive control resources. Although memory complaints have been reported among Egyptian university students, the association between ER strategies and SMP remains largely unexplored. This study aimed to assess ER strategies and SMP among undergraduate students at Port Said University and examine the association between these constructs.

**Methods:**

A descriptive cross-sectional study with an analytical component was conducted among 1,287 undergraduate students from seven randomly selected faculties at Port Said University. Data were collected using a self-administered questionnaire comprising the Emotion Regulation Questionnaire (ERQ) to assess CR and ES and the PRMQ to measure SMP. Data were analyzed using Jamovi version 2.3.28 employing descriptive statistics, nonparametric tests, Spearman’s rank correlation, and multiple linear regression.

**Results:**

Participants had a median total ERQ score of 44 and a median total PRMQ score of 50. Males reported significantly higher ES and total ER scores than females (*p* < 0.001 and *p* = 0.005, respectively), whereas females reported significantly higher PRMQ scores (*p* < 0.001). Faculty affiliation was significantly associated with both total ERQ and total PRMQ scores, whereas academic grade was associated only with total ERQ scores and family income only with total PRMQ scores. All ERQ subscales were significantly and negatively correlated with PRMQ scores (all *p* < 0.001). The largest correlation coefficient was observed between total ERQ and total PRMQ scores (ρ = −0.226), indicating a weak inverse association. In multiple linear regression adjusting for sex, faculty, and family income, both CR (B = − 0.272, 95% CI: −0.360 to − 0.184) and ES (B = − 0.270, 95% CI: −0.391 to − 0.150) were independently and inversely associated with PRMQ scores (both *p* < 0.001).

**Conclusion:**

The findings suggest that greater reported use of ER strategies is associated with fewer self-reported memory complaints among undergraduate students. Although the association was statistically significant, its magnitude was weak, indicating that ER represents only one of several factors associated with SMP. This suggests that effective emotional processing may support the cognitive resources required for daily memory tasks.

**Supplementary Information:**

The online version contains supplementary material available at https://doi.org/10.1186/s40359-026-05679-9.

## Background

Emotion regulation (ER) encompasses the mechanisms by which individuals observe, assess, and alter their emotional responses to pursue goals, and it plays a crucial role in mental health and overall emotional well-being. The habitual reliance on different ER strategies is associated with distinct long-term psychological outcomes. Cognitive reappraisal (CR), an antecedent-focused strategy that involves reframing the meaning of emotionally evocative situations, is consistently linked to adaptive psychological functioning. Conversely, expressive suppression (ES), a response-focused strategy characterized by the suppression of emotional expression, has been related to maladaptive outcomes, including elevated affective symptoms. Understanding the ER tendencies of populations such as university undergraduates is therefore important for improving our understanding of factors associated with cognitive and emotional resilience [1–4]. 

ER strategies have been linked to higher-order cognitive functioning, providing a conceptual basis for examining their association with subjective memory performance (SMP). Both processes have been proposed to rely on shared, capacity-limited executive control resources mediated by the prefrontal cortex. While CR has been proposed to conserve cognitive resources by reducing emotional interference, ES has been proposed to require greater cognitive resources due to its reliance on continuous monitoring and inhibition. Accordingly, habitual reliance on ES has been hypothesized to contribute to reduced efficiency in complex cognitive processes, including those involved in prospective memory. Consequently, individual differences in ER strategy use may correspond to variations in the cognitive resources available for everyday memory tasks and, therefore, with differences in SMP [5]. 

SMP refers to an individual’s perception of their everyday memory functioning. Subjective Memory Complaints (SMCs) represent one manifestation of poorer subjective memory performance and are commonly assessed in population-based studies. SMP reflects an individual’s self-assessment of the frequency and impact of everyday memory lapses. Although not always perfectly correlated with objective performance, SMP is a clinically significant indicator: it is a robust predictor of future cognitive decline and a crucial index of cognitive self-awareness and emotional health. Contemporary research suggests that subjective memory questionnaires are best understood as unidimensional instruments, capturing a generalized perception of forgetfulness across both prospective (future-oriented) and retrospective (past-oriented) domains. Given the reported associations between specific ER strategies and cognitive functioning, SMP may reflect not only objective memory ability but also differences in individuals’ ER strategies and related cognitive processes [6–10]. 

Studies conducted among Egyptian students have reported distinct psychosocial correlates of habitual ER. While CR has consistently been linked to positive social functioning, ES has consistently been linked to less favorable interpersonal outcomes. The reliability and validity of these ER constructs have been established through research among Egyptian adolescents in Nasr City. The distinct ER strategies carry significant implications for affective and social functioning. [1, 4] Concurrently, research among final-year medical students at Mansoura University found that 62.3% of students experienced SMCs, with 57.6% of them characterizing their memory as “fairly good with occasional lapses” [11]. 

ER strategies, particularly CR and ES, have been consistently associated with students’ psychological well-being and cognitive functioning. Likewise, SMP is an important indicator of perceived cognitive strain and is strongly linked to stress and negative affect in young adults. Although Egyptian studies have reported high prevalence of SMP concerns and confirmed the validity of ER measures, no study has examined patterns of ER strategy use among undergraduate students in Egypt. Furthermore, the association between ER strategies and SMP among Egyptian undergraduate students remains largely unexplored, and international studies examining this association are limited, despite evidence suggesting that both are related to shared executive control processes. Addressing this gap may improve understanding of the association between ER strategies and SMP in university students. Therefore, this study aimed to investigate the association between emotion regulation strategies and subjective memory performance among undergraduate students at Port Said University. Based on previous literature, we hypothesized that greater use of emotion regulation strategies would be associated with better subjective memory performance, with cognitive reappraisal demonstrating a stronger beneficial association than expressive suppression.

## Methodology

### Study design

A descriptive cross-sectional study design with an analytic component was selected to assess emotion regulation and subjective memory performance among students and to examine their association.

### Study population and setting

The study was conducted between December 2025 and April 2026 among undergraduate students at Port Said University using a validated self-administered questionnaire. Seven faculties (Engineering, Medicine, Science, Specific Education, Law, Management Technology and Information Systems (MTIS), and Physical Therapy) were selected using simple random sampling through the RAND function in Microsoft Excel (Microsoft 365 MSO Version 2511).

### Sample size

The sample size was estimated using the single population proportion formula ($\:n=\:\frac{{{(Z}_{1-\alpha\:/2})}^{2}\:P(1-P)}{{d}^{2}})$, based on a previously reported prevalence of SMCs among Egyptian medical students (62.3%), a 95% confidence level and a 5% margin of error, yielding a sample of 361 participants. To account for an anticipated 10% non-response rate, the sample size was adjusted using the formula ($\:{n}_{adjusted}=\frac{n}{1-r}$), where * r=0.10*, resulting in a minimum required sample size of 402 participants. A total of 1,287 eligible participants completed the survey during the predefined data collection period, exceeding the minimum required sample size and thereby improving the precision of the study estimates. [11, 12]

### Sample Procedure

A two-stage sampling approach was employed. First, seven faculties were selected using simple random sampling. Second, eligible undergraduate students within the selected faculties were recruited using convenience sampling until the end of the predefined data collection period. A total of 1,365 students were approached in common areas and after lectures during the study period. The study objectives were explained, participation was voluntary, and questionnaires were distributed to students who agreed to participate and provided informed consent. Of the 1,365 students approached, 1,287 completed the questionnaire, yielding a response rate of 94.28%.

- **Inclusion criteria**: Undergraduate students enrolled at Port Said University who were present during the data collection period and provided informed consent.
- **Exclusion criteria**: Students who declined participation or submitted incomplete questionnaires.

### Data collection tool

Data were collected through an Arabic, self-administered, validated, structured questionnaire designed to measure ER strategies and SMP among Port-Said University undergraduate students. Full detailed questionnaire available in supplementary file 1.

The questionnaire consisted of three main sections:

#### **Sociodemographic data **

This section collected sociodemographic characteristics of participants, including age, sex, nationality, faculty, academic year, grade, smoking status, caffeine intake, residence, and household income

#### Emotion Regulation Questionnaire (ERQ)

An Arabic version of the 10-item self-report instrument, validated by Kahwagi et al. (2021), was used to assess two emotion regulation strategies: Cognitive Reappraisal (CR; items 1, 3, 5, 7, 8, and 10) and Expressive Suppression (ES; items 2, 4, 6, and 9). Each item is rated on a 7-point Likert scale ranging from 1 (strongly disagree) to 7 (strongly agree), with no reverse-scored items. Scores for each subscale were calculated by summing the corresponding items, while a total ERQ score was obtained by summing all items (range: 10–70). Higher scores indicate greater self-reported use of emotion regulation strategies (CR and ES). No established cut-off values are available; therefore, scores were analyzed as continuous variables. In the present study, Cronbach’s α coefficients were 0.756 for the ERQ total scale, 0.748 for CR, and 0.668 for ES [2].

#### Prospective and Retrospective Memory Questionnaire (PRMQ)

A validated Arabic version of the 16-item PRMQ, originally developed by Smith et al. (2000), was used to assess the frequency of self-reported everyday memory failures as an indicator of SMP. Each item is rated on a 5-point Likert scale ranging from 1 (never) to 5 (very often), with no reverse scoring. The items are grouped into eight memory categories based on time frame (short-term vs. long-term), memory type (prospective vs. retrospective), and cue source (self-cued vs. environmentally cued), with two items per category. Total scores range from 16 to 80, with higher scores indicating poorer SMP. The questionnaire does not have established cut-off values; therefore, scores were analyzed as continuous variables. In the present study, Cronbach’s α for the PRMQ was 0.864 [13].

### Data management

Data analysis was performed using Microsoft Excel 365 for data cleaning and Jamovi version 2.3.28 for statistical analysis. The normality of the data was tested using the Shapiro-Wilk test, which indicated a non-normal distribution requiring non-parametric tests. Frequencies and percentages were used to describe qualitative variables and categorical outcomes, while the median, first and third quartiles (Q1, Q3) were used to describe quantitative variables. Statistical significance was tested using the Mann-Whitney U test and the Kruskal-Wallis test, with a Dwass-Steel-Critchlow-Fligner (DSCF) pairwise comparison utilized for post-hoc analysis following the Kruskal-Wallis test. Spearman’s rank correlation coefficient (ρ) was used to assess associations between continuous variables.

Multiple linear regression was used to examine the independent associations. Although the Shapiro–Wilk test indicated non-normality of the raw scores, linear regression assumes normality of residuals rather than of the outcome variable; this assumption was therefore assessed directly. Linearity and homoscedasticity were evaluated by inspection of residuals against fitted values and by the Breusch–Pagan, Goldfeld–Quandt, and Harrison–McCabe tests; normality of residuals by the Shapiro–Wilk test and a normal Q–Q plot; multicollinearity by variance inflation factors (VIF); and influential observations by Cook’s distance. The difference between the CR and ES coefficients was tested using a Wald test for the linear contrast between them. Coefficients are reported as unstandardized B with 95% confidence intervals.

The level of significance was set at *p* < 0.05.

### Ethical Considerations

This study was approved by the Ethical Committee at the Faculty of Medicine, Port Said University (ERN number: MED (1/2/2026) s.no (377) PHI_021). The research was carried out in agreement with the standards of the Helsinki Declaration. All participants provided informed consent before participation.

## Results

### Sociodemographic characteristics

The study included 1,287 participants. The median age was 20 years (IQR: 19–21) with nearly equal sex distribution. Most participants were Egyptian (95.2%) and urban residents (81.3%). Medical students formed the largest faculty group (26.2%), and first- and second-year students comprised the majority. Most reported very good or excellent grades (81.8%). The majority were non-smokers (92.5%) and consumed caffeine regularly while over half reported that their family income was sufficient to meet routine and emergency needs (51.6%), as shown in Table1.

**Table 1:** Sociodemographic characteristics of the study participants (N = 1,287)

		Frequency		Percentage				
**Age**	Median (Q1, Q3)	20 (19, 21)						
**Sex**	Male	613		47.6%				
	Female	674		52.4%				
**Nationality**	Egyptian	1225		95.2%				
	Non-Egyptian	62		4.8%				
**Faculty**	Engineering	172		13.4%				
	Medicine	337		26.2%				
	Science	149		11.6%				
	MTIS	118		9.2%				
	Physical Therapy	94		7.3%				
	Specific Education	227		17.6%				
	Law	190		14.8%				
**Academic year**	First	412		32.0%				
	Second	387		30.1%				
	Third	295		22.9%				
	Fourth	151		11.7%				
	Fifth	42		3.3%				
**Academic grade**	Excellent	490		38.1%				
	Very good	563		43.7%				
	Good	188		14.6%				
	Pass	39		3.0%				
	Fail	7		0.5%				
**Smoking status**	Non-smoker	1191		92.5%				
	Current smoker	59		4.6%				
	Ex-smoker*	37		2.9%				
**Caffeine intake**	None	225		17.5%				
	Coffee	340		26.4%				
	Tea	373		28.9%				
	Others	89		6.9%				
	More than one	261		20.3%				
**Residence**	Rural	241		18.7%				
	Urban	1046		81.3%				
**Family income**	In debt	58		4.5%				
	Meet routine needs	375		29.1%				
	Meet routine and emergency needs	664		51.6%				
	Save and invest	190		14.8%				

Q1: First Quartile

Q3: Third Quartile

MTIS: Management Technology and Information Systems

### Emotion regulation

Median ERQ item scores ranged from 3 to 5. The CR subscale had a median score of 27 (Q1 = 22, Q3 = 32), while the ES subscale showed a median of 17 (Q1 = 13, Q3 = 21). The overall median ERQ score was 44 (Q1 = 37, Q3 = 51), as shown in Table 2. Detailed item scores are available in Supplementary file 2.

**Table 2:** Median (Q1–Q3) scores of the Emotion Regulation Questionnaire (ERQ)

			Quartiles	
	Possible score	Median	Q1	Q3
Total:				
Total score:	10-70	44	37	51
Total CR score:	6-42	27	22	32
Total ES score:	4-28	17	13	21

Q1: First Quartile

Q3: Third Quartile

CR: Cognitive Reappraisal

ES: Expressive Suppression

### Subjective memory performance

The PRMQ responses showed variation in the frequency of self-reported memory lapses. The prospective memory subscale showed slightly higher scores compared to retrospective subscales of median (26 and 24 respectively). The overall PRMQ total score had a median of 50 (Q1 = 43, Q3 = 58.5), as detailed in Table 3. Detailed item scores are available in Supplementary file 2.

**Table 3:** Median (Q1–Q3) scores of the Prospective-Retrospective Memory Questionnaire (PRMQ)

			Quartiles	
	Possible score	Median	Q1	Q3
Total:				
Total Prospective score	8–40	26	21	30
Total Retrospective score	8–40	24	20	29
Total score	16–80	50	43	58.50

Q1: First Quartile

Q3: Third Quartile

### Association between sociodemographic characteristics and emotion regulation

Sociodemographic characteristics showed several significant associations with ERQ scores. Sex was significantly related to ES and total ERQ, with males scoring higher (*p* < 0.001, *p* = 0.005). Faculty was significantly associated with CR (*p* = 0.034), ES (*p* = 0.004), and total ERQ (*p* = 0.016), with post hoc differences in CR between Engineering vs. Specific Education (*p* = 0.035) and Physical Therapy vs. Specific Education (*p* = 0.042), and in ES between Engineering vs. Science (*p* = 0.015); total ERQ also differed between Physical Therapy vs. Specific Education (*p* = 0.013) and Science vs. Specific Education (*p* = 0.039). Academic grade showed significant associations with CR (*p* = 0.037) and total ERQ (*p* = 0.047), with post hoc differences in CR (very good vs. pass, *p* = 0.023) and in total ERQ (very good vs. pass, *p* = 0.040). Family income was significantly associated with CR (*p* = 0.019), with post hoc differences between in debt vs. meet routine needs (*p* = 0.036), in debt vs. meet routine and emergency needs (*p* = 0.025), and in debt vs. save and invest (*p* = 0.015). Residence was also significantly associated with ES (*p* = 0.042), as shown in Table 4.

**Table 4:** Association between (ERQ) and sociodemographic characteristics of the study participants

		Cognitive reappraisal		Expressive suppression		Total ERQ Score	
		Median (Q1, Q3)	*p*-value (ɛ^2^)	Median (Q1, Q3)	*p*-value (ɛ^2^)	Median (Q1, Q3)	*p*-value (ɛ^2^)
Sex	Male	27 (22, 32)	0.500^a^ (0.022)	18 (14, 22)	< 0.001* ^**a**^ (0.197)	45 (37, 52)	0.005* ^**a**^ (0.091)
	Female	28 (22, 33)		16 (12, 20)		43 (36, 50)	
Nationality	Egyptian	27 (22, 32)	0.315^a^ (0.076)	17 (13, 21)	0.657^a^ (0.033)	44 (37, 51)	0.411^a^ (0.062)
	Non-Egyptian	27 (21, 31.8)		16.5 (12, 21)		43.5 (35.3, 51.5)	
Faculty	Engineering	26 (22, 31.3)	0.034* ^**b**^ (0.011)	17 (14, 22.3)	0.004* ^**b**^ (0.015)	44 (36, 50.3)	0.016* ^**b**^ (0.012)
	Medicine	27 (22, 33)		17 (13, 21)		44 (37, 51)	
	Science	27 (23, 32)		16 (11, 19)		42 (36, 49)	
	MTIS	27 (23, 33)		17 (13, 22)		46 (37, 52)	
	Physical Therapy	26 (20.3, 31)		16 (12, 20)		41.5 (33.3, 47.8)	
	Specific Education	29 (24, 33)		17 (13, 20.5)		46 (40, 51)	
	Law	28 (21.3, 33)		16 (12, 20)		44 (35.3, 52)	
Academic year	First	28 (23, 33)	0.287^b^ (0.004)	17 (13, 21)	0.548^b^ (0.002)	45 (38, 51)	0.319^b^ (0.004)
	Second	27 (21, 32)		16 (12, 21)		43 (35, 51)	
	Third	27 (22, 32.5)		16 (12, 20)		44 (36.5, 51)	
	Fourth	28 (22, 32)		17 (14, 21)		45 (38, 52)	
	Fifth	27.5 (23.3, 32)		16 (12.3, 20.8)		43.5 (36.3, 49)	
Academic grade	Excellent	27 (22, 33)	0.037* ^**b**^ (0.008)	16 (12, 20)	0.165^b^ (0.005)	44 (36, 50)	0.047* ^**b**^ (0.008)
	Very good	28 (22, 33)		17 (13, 21)		45 (37, 52)	
	Good	27 (22, 32)		17 (14, 21)		44 (36, 50.3)	
	Pass	23 (18.5, 27)		15 (12, 19)		39 (32, 47)	
	Fail	22 (19.5, 30)		16 (12, 18.5)		34.5 (33, 48.5)	
Smoking status	Non-smoker	28 (22, 32)	0.163^b^ (0.003)	17 (13, 21)	0.797^b^ (0.000)	44 (37, 51)	0.647^b^ (0.001)
	Smoker	25 (20, 30.5)		17 (12.5, 21.5)		43 (35, 50.5)	
	Ex-smoker	27 (21, 33)		17 (14, 21)		45 (35, 54)	
Caffeine intake	None	28 (22, 32)	0.471^b^ (0.003)	17 (13, 21)	0.892^b^ (0.001)	45 (37, 52)	0.759^b^ (0.002)
	Coffee	26.5 (21, 33)		17 (12, 21)		43 (35, 52.3)	
	Tea	28 (23, 33)		17 (13, 20)		45 (37, 51)	
	Other	28 (23, 33)		17 (14, 21)		45 (40, 52)	
	More than one	27 (22, 32)		16 (12, 20)		43 (37, 50)	
Residence	Rural	28 (22, 33)	0.705^a^ (0.016)	18 (13, 22)	0.042* ^**a**^ (0.084)	46 (36, 52)	0.201^a^ (0.053)
	Urban	27 (22, 32)		16 (13, 20)		44 (37, 51)	
Family income	In debt	23 (18, 30)	0.019* ^**b**^ (0.008)	15.5 (12, 20.8)	0.472^b^ (0.002)	39.5 (33, 48)	0.078^b^ (0.005)
	Meet routine needs	27 (22, 32)		17 (13, 21)		44 (37, 51)	
	Meet routine and emergency needs	27 (22, 32)		17 (13, 20)		44 (37, 51)	
	Save and invest	29 (23, 33)		17 (11.3, 20.8)		45 (37, 51.8)	

ɛ^2^: Effect size, a: Mann-Whitney U test, b: Kruskal-Wallis test, *: Level of significance at *p* < 0.05.

ERQ: Emotion Regulation Questionnaire

Q1: First Quartile

Q3: Third Quartile

MTIS: Management Technology and Information Systems

### Association between sociodemographic characteristics and subjective memory performance

Sociodemographic characteristics showed significant associations with SMP across multiple domains. Sex was highly significant for Prospective, Retrospective, and total PRMQ scores (all *p* < 0.001), and faculty was also associated with all domains (all *p* ≤ 0.001). Post hoc analysis revealed differences in Prospective memory between MTIS vs. Law (*p* = 0.049), Physical Therapy vs. Law (*p* = 0.028), and Specific Education vs. Law (*p* < 0.001); in Retrospective memory between Law vs. Science (*p* = 0.026), Law vs. Physical Therapy (*p* = 0.044), and Law vs. Specific Education (*p* = 0.005); and in total PRMQ scores between Medicine vs. Specific Education (*p* = 0.031), Physical Therapy vs. Law (*p* = 0.016), and Specific Education vs. Law (*p* < 0.001). In addition, family income was significantly associated with Retrospective memory (*p* = 0.034) and total PRMQ scores (*p* = 0.021), with post hoc differences observed between the in debt vs. save and invest categories for both domains (*p* = 0.038 and *p* = 0.023, respectively), as presented in Table 5.

**Table 5:** Association between (PRMQ) and sociodemographic characteristics of the study participants

		Prospective memory		Retrospective memory		Total PRMQ Score	
		Median (Q1, Q3)	*p*-value (ɛ^2^)	Median (Q1, Q3)	*p*-value (ɛ^2^)	Median (Q1, Q3)	*p*-value (ɛ^2^)
Sex	Male	25 (20, 29)	< 0.001* ^**a**^ (0.149)	24 (20, 28)	< 0.001* ^**a**^ (0.145)	48 (42, 56)	< 0.001* ^**a**^ (0.159)
	Female	26 (22, 31)		25 (21, 30)		52 (44, 60)	
Nationality	Egyptian	26 (21, 30)	0.433^a^ (0.059)	24 (20, 29)	0.562^a^ (0.044)	50 (43, 58)	0.892^a^ (0.010)
	Non-Egyptian	27 (22, 30)		23 (20, 29)		50 (43.3, 59.8)	
Faculty	Engineering	26 (21, 30)	< 0.001* ^**b**^ (0.018)	25 (21, 28.3)	0.001* ^**b**^ (0.017)	51 (43, 58)	< 0.001* ^**b**^ (0.019)
	Medicine	25 (21, 30)		24 (20, 28)		49 (42, 58)	
	Science	25 (22, 31)		25 (21, 30)		51 (43, 61)	
	MTIS	26.5 (21.3, 30)		24 (20, 29.8)		50.5 (42, 60)	
	Physical Therapy	26 (23, 30)		25.5 (22, 29)		51 (47, 58.8)	
	Specific Education	26 (23, 31)		26 (22, 29)		52 (46, 60)	
	Law	24 (20, 28)		23 (18, 27)		47.5 (38.3, 55.8)	
Academic year	First	26 (22, 30)	0.524^b^ (0.003)	25 (21, 29)	0.097^b^ (0.006)	51 (44, 58)	0.281^b^ (0.004)
	Second	25 (21, 30)		25 (21, 29.5)		50 (43, 59)	
	Third	25 (21, 30)		24 (20, 28)		49 (41, 58)	
	Fourth	25 (22, 30)		23 (21, 29)		48 (43, 58)	
	Fifth	28.5 (22.3, 31)		25.5 (21.3, 28)		53 (44.3, 58.8)	
Academic grade	Excellent	26 (22, 30)	0.353^b^ (0.003)	24.5 (21, 29)	0.078^b^ (0.007)	50 (44, 59)	0.115^b^ (0.006)
	Very good	26 (21, 30)		24 (20, 28)		50 (42, 58)	
	Good	24.5 (21, 30)		24 (20, 29)		48 (43, 58.3)	
	Pass	28 (24, 32)		26 (22.5, 29)		54 (48, 59.5)	
	Fail	30 (26, 31.5)		32 (29, 34)		62 (56.5, 65)	
Smoking status	Non-smoker	26 (21, 30)	0.425^b^ (0.001)	24 (20, 29)	0.653^b^ (0.001)	50 (43, 58.5)	0.495^b^ (0.001)
	Smoker	25 (21.5, 29)		25 (20, 28)		49 (43, 55)	
	Ex-smoker	26 (23, 31)		25 (21, 31)		50 (45, 61)	
Caffeine intake	None	25 (20, 30)	0.186^b^ (0.005)	24 (20, 29)	0.089^b^ (0.006)	48 (42, 58)	0.067^b^ (0.007)
	Coffee	26 (21, 30)		25 (21, 29)		51 (43, 58.3)	
	Tea	26 (22, 30)		25 (21, 29)		51 (44, 58)	
	Other	26 (23, 30)		25 (21, 30)		52 (47, 60)	
	More than one	25 (21, 30)		24 (20, 28)		48 (42, 58)	
Residence	Rural	26 (21, 30)	0.298^a^ (0.043)	24 (20, 29)	0.848^a^ (0.008)	50 (43, 59)	0.669^a^ (0.018)
	Urban	26 (21, 30)		24.5 (20, 29)		50 (43, 58)	
Family income	In debt	27 (24, 32)	0.054^b^ (0.006)	27 (22, 32)	0.034* ^**b**^ (0.007)	54 (48, 63.5)	0.021* ^**b**^ (0.008)
	Meet routine needs	26 (22, 30)		25 (21, 28)		51 (43, 58)	
	Meet routine and emergency needs	26 (21, 30)		24 (20, 29)		50 (43, 59)	
	Save and invest	25 (21, 29)		24 (19, 28)		49 (40, 56)	

ɛ^2^: Effect size, a: Mann-Whitney U test, b: Kruskal-Wallis test, *: Level of significance at *p* < 0.05.

PRMQ: Prospective, Retrospective Memory Questionnaire

Q1: First Quartile

Q3: Third Quartile

MTIS: Management Technology and Information Systems

### Association between emotion regulation and subjective memory performance

**Fig. 1:**
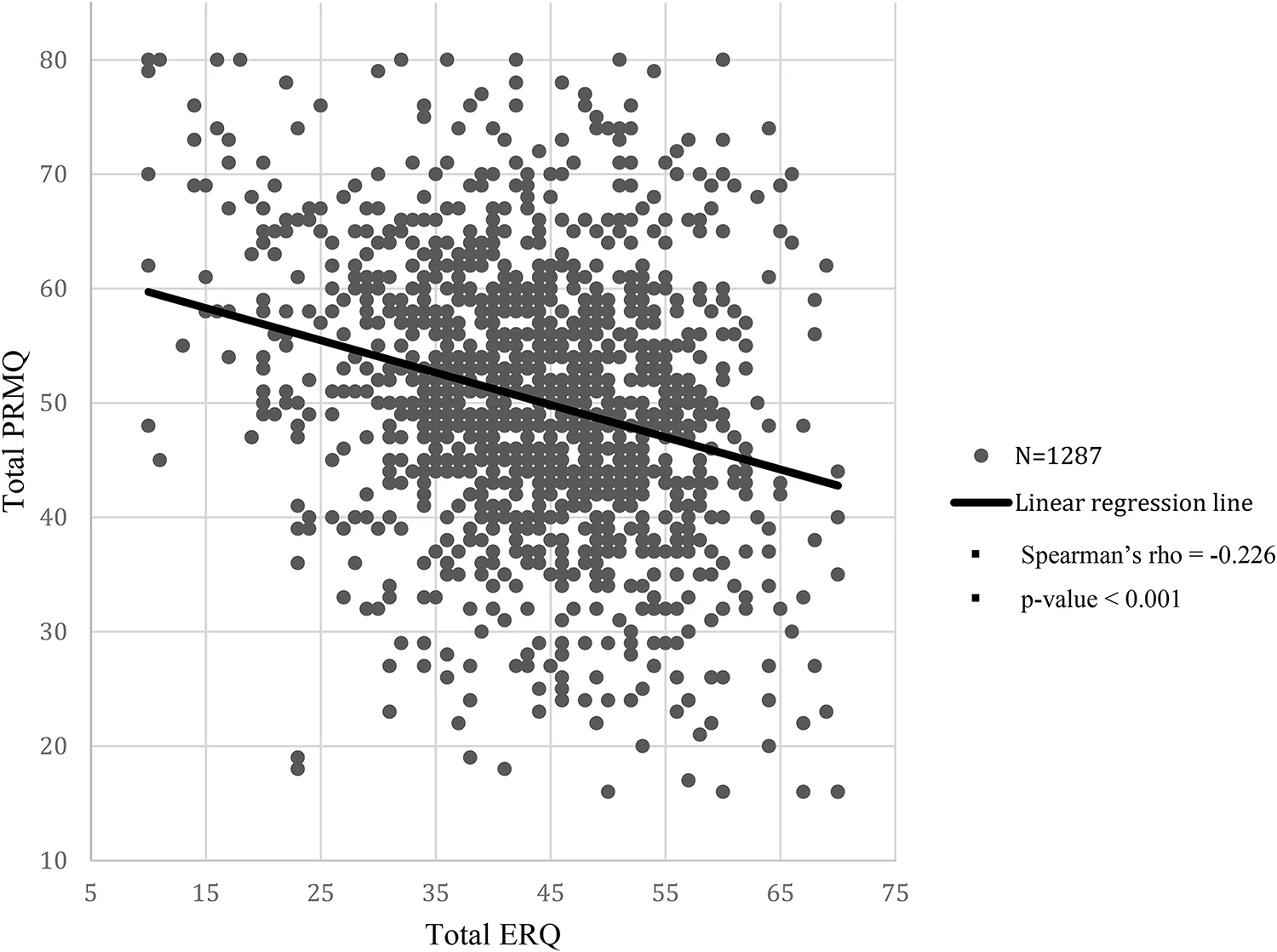
Correlation between total ERQ and total PRMQ scores (*N* = 1,287). The fitted line is shown for visual illustration only; reported associations are based on Spearman’s ρ

Table 6 demonstrates a statistically significant, negative correlation between all ERQ subscales and PRMQ domains (*p* < 0.001). Spearman’s rho values indicate weak to moderate associations (ρ = -0.153 to -0.226), suggesting that greater use of ER strategies, is consistently associated with better SMP across prospective and retrospective memory domains. The largest correlation coefficient was observed between total ERQ and total PRMQ scores (ρ = −0.226). Figure 1 illustrates the correlation between total ERQ and total PRMQ scores. The distribution of data points shows a slight downward trend, indicating a negative association between ER and self-reported memory complaints.

**Table 6:** Correlations between ERQ and PRMQ subscale and total scores

		CR ERQ	ES ERQ	Total ERQ
** Prospective**	Spearman’s rho	-0.178	-0.153	-0.198
	*p*-value	< 0.001	< 0.001	< 0.001
**Retrospective**	Spearman’s rho	-0.194	-0.176	-0.221
	p-value	**< 0.001**	**< 0.001**	**< 0.001**
**Total PRMQ**	Spearman’s rho	-0.201	-0.177	-0.226
	p-value	**< 0.001**	**< 0.001**	**< 0.001**

ERQ: Emotion Regulation Questionnaire

PRMQ: Prospective, Retrospective Memory Questionnaire

CR: Cognitive Reappraisal

ES: Expressive Suppression

### Multivariable analysis of factors associated with total PRMQ score

A multiple linear regression was conducted to examine the associations between SMP, as assessed by PRMQ scores, and CR and ES, as well as sex, academic faculty, and family income (Table 7). The model accounted for approximately 10.2% of the variance (R² = 0.102, Adjusted R² = 0.093).

**Table 7:** Multiple linear regression analysis of factors associated with total PRMQ score

Predictor		B	SE	95% CI	β	*p*
				[LL, UL]		
Intercept		63.55	2.05	[59.52, 67.58]		< 0.001*
Cognitive reappraisal		-0.272	0.045	[-0.36, -0.18]	-0.173	< 0.001*
Expressive suppression		-0.270	0.061	[-0.39, -0.15]	-0.127	< 0.001*
Sex	Male vs. Female	-2.812	0.727	[-4.23, -1.39]	-0.235	< 0.001*
Faculty	Engineering vs. Law	4.079	1.228	[1.67, 6.49]	0.340	< 0.001*
	Medicine vs. Law	2.973	1.059	[0.90, 5.05]	0.248	0.005*
	Science vs. Law	3.539	1.256	[1.07, 6.00]	0.295	0.005*
	MTIS vs. Law	4.210	1.340	[1.58, 6.84]	0.351	0.002*
	Physical Therapy vs. Law	4.993	1.447	[2.15, 7.83]	0.417	< 0.001*
	Specific Education vs. Law	4.849	1.143	[2.61, 7.09]	0.405	< 0.001*
Family income	Meet routine needs vs. In debt	-2.974	1.620	[-6.15, 0.20]	-0.248	0.067
	Meet routine & emergency needs vs. In debt	-3.813	1.581	[-6.91, -0.71]	-0.318	0.016*
	Save & invest vs. In debt	-4.436	1.729	[-7.82, -1.04]	-0.370	0.010*

R^2^ = 0.102, Adjusted R^2^ = 0.093, *N* = 1,287, Reference category for Sex = Female, Reference category for Faculty = Law, Reference category for Family = In debt. B = unstandardized coefficient, CI = confidence interval; LL = lower limit; UL = upper limit, β = standardized coefficient; expressed in outcome SD units for categorical predictors and therefore not comparable with values for continuous predictors. * Level of significance at *p* < 0.05. PRMQ: Prospective and Retrospective Memory Questionnaire, MTIS: Management Technology and Information Systems

Both ER subscales were significantly and negatively associated with PRMQ scores, with each one-point increase corresponding to a 0.272-point lower score for CR (95% CI: −0.360 to − 0.184) and a 0.270-point lower score for ES (95% CI: −0.391 to − 0.150), both *p* < 0.001. The two coefficients did not differ significantly (B difference = − 0.002, *p* = 0.98), indicating comparable independent associations between the two strategies. Higher ER scores were therefore associated with fewer self-reported memory difficulties and, accordingly, better SMP.

Males had significantly lower PRMQ scores than females (B = − 2.812, 95% CI: −4.238 to − 1.385, *p* < 0.001), and students in all non-law faculties scored higher than those in Law (all *p* ≤ 0.005), with the largest difference observed for Physical Therapy (B = 4.993, 95% CI: 2.154 to 7.832). The overall effect of family income did not reach statistical significance (*p* = 0.051); individual contrasts suggested lower scores among more financially secure students relative to those in debt, but this should be interpreted with caution.

Residual plots showed constant spread across fitted values with no evidence of non-linearity, and residuals were approximately normally distributed (Shapiro–Wilk *p* = 0.115), with no multicollinearity (all VIF ≤ 1.14) or influential observations (maximum Cook’s distance = 0.018). The Breusch–Pagan test was statistically significant (*p* = 0.005), although the Goldfeld–Quandt (*p* = 0.173) and Harrison–McCabe (*p* = 0.204) tests were not; given the large sample size, this likely reflects the sensitivity of the Breusch–Pagan test rather than a meaningful violation.

## Discussion

This study is among the first to provide a comprehensive analysis of the association between ER and SMP using validated tools among a robust sample of 1,287 Egyptian undergraduate students across multiple diverse faculties. The data indicate a statistically significant negative correlation between all ERQ subscales and PRMQ domains, even though it is considered weak. This finding suggests that individuals who report more frequent use of either ER strategy report fewer daily perceived memory lapses. Contrary to our hypothesis, CR was not more strongly associated with memory complaints than ES. This pattern is consistent with the proposal that emotional management and memory processes draw upon shared, capacity-limited executive control resources mediated by the prefrontal cortex.

While these correlations are highly statistically significant due to our robust sample size, the modest correlation coefficients suggest that ER is one of several factors associated with perceived memory. From a practical perspective, however, ER represents a highly modifiable target for behavioral interventions.

The present study investigated habitual use of ER strategies, specifically CR and ES, yielding median scores of 27 for CR and 17 for ES. These findings are broadly comparable to reported values across diverse international contexts. For instance, while a United States general community sample demonstrated a higher mean for CR (28.78) and a lower mean for ES (15.04) [3], our Egyptian cohort exhibited a comparatively higher tendency toward suppression. This elevation in ES likely reflects specific Middle Eastern cultural paradigms, where emotional restraint is frequently prioritized to preserve social harmony [14]. Indeed, the present findings closely mirror those of a Lebanese adult sample, which reported strikingly similar means (CR ≈ 26.5; ES ≈ 17), suggesting robust regional consistency [2]. Furthermore, evidence from Saudi Arabian and Iranian student populations corroborates this regional profile, demonstrating moderate engagement in both strategies. [15, 16]

Moving beyond regional variations, the current study’s analyses demonstrated significant associations between sociodemographic characteristics and ER preferences. Corroborating findings from comparable studies in Saudi Arabia, Iran, and Mexico, sex was identified as a primary factor associated with ER preference. Specifically, males in the current cohort showed a significantly greater reliance on ES than females. This closely mirrors the results of the Iranian study, which reported a similar male propensity for ES. This cross-cultural alignment indicates a pervasive tendency among men to inhibit outward emotional displays. Additionally, this aligns with broader regional literature, such as the Saudi Arabian study, which found that females employ CR more frequently than their male counterparts (mean = 3.17 vs. 2.83, respectively) [15–17]. 

Academic achievement and socioeconomic status emerged as significant contextual correlates of ER. Higher academic grades were positively associated with CR, consistent with UAE-based findings, in which habitual reappraisal predicted enhanced well-being. However, unlike the UAE study, which found that chronic suppression negatively predicted academic performance, we detected no significant association between suppression and academic performance [18]. Financial stress similarly appeared to constrain adaptive regulatory capacity. Although income has often been treated as a peripheral variable in regional studies from Lebanon and Pakistan, [2, 19] the current findings align with Chilean evidence showing that a negative problem-solving orientation was associated with significantly higher depressive symptoms in a low-income antenatal sample, highlighting a critical link between socioeconomic security and regulatory capacity [20]. 

Finally, variance in ER was observed across specific academic disciplines (e.g., Specific Education, Engineering, Physical Therapy), suggesting that distinct educational environments may subtly shape how students manage stress, providing a counter-narrative to previous studies that found no distinction between STEM and non-STEM cohorts [15]. 

While the present study focused on demographic correlates of ER strategies, these findings operate within a broader framework linking ER strategies to psychological health. For context, clinical research in Lebanon, Egypt, and the US has established that higher anxiety levels are significantly associated with diminished CR, [2, 21, 22] whereas ES consistently correlates with psychological distress and depression. [23, 24]

The current study observed a median PRMQ score of 50 among Egyptian undergraduates, which was higher than scores reported in several comparable populations. A large sample of 297,242 adults, reported by researchers at the University of Edinburgh, UK, had a mean of 40.39 [6], while healthy Portuguese young adults averaged 39.10 [10]. Even older populations scored lower, as seen in a Finnish cohort with a mean of 36.3 [25]. This pattern suggests that the Egyptian undergraduate cohort reported more frequent everyday memory failures than those reported in these international samples, indicating comparatively poorer SMP.

This elevation may be relevant to academic and psychosocial pressures characteristic of undergraduate life. Research from Spain in 2021 indicates that SMCs occur at comparable rates in young and older adults but are more strongly tied to emotional distress in younger groups [7]. This is mirrored in recent local Egyptian evidence, where 62.3% of Mansoura University medical students reported poorer SMP, and 27.8% of Horus University undergraduates demonstrated inadequate memory skills in 2023 and 2025, respectively. Across these studies, subjective memory difficulties were associated with factors such as anxiety, depression, sleep deprivation, and excessive social media use, suggesting that psychological and lifestyle factors may be relevant to individual differences in SMP. Although these factors have been proposed to influence attention, memory encoding, and perceived memory functioning, the mechanisms underlying these associations cannot be established from the present cross-sectional findings. [11, 26]

Examining memory subtypes, prospective memory complaints were slightly higher than retrospective ones, consistent with the Portuguese young adult literature [10]. On the other hand, the Finnish study revealed that older adults tend to show a more balanced pattern across both domains, likely reflecting reduced prospective demands following academic and professional retirement [25]. Notably, while student complaints appear rooted in stress and cognitive overload, elderly populations tell a different story; a Korean cohort in which approximately 39% reported complaints showed stronger links to measurable neurocognitive decline [27]. 

Demographic variables further shaped complaint levels. While some broad literature argues that sex does not play a prominent role in general memory complaints [28], females reported more complaints than males, consistent with prior young adult findings, likely reflecting greater anxiety sensitivity among women [10]. Financial status emerged as another meaningful factor, with students reporting economic strain showing higher complaint levels compared to financially secure peers.

This study also demonstrates that greater use of both CR and ES was associated with fewer subjective memory concerns, corresponding to better SMP, challenging the typical assumption that suppression may be associated with greater cognitive costs. However, the supportive association between CR and memory performance perfectly aligns with broader findings in cognitive psychology. It has been emphasized that CR functions as a proactive strategy, facilitating more efficient encoding and retrieval of emotional information [29]. Furthermore, it has been posited that these adaptive ER strategies are essential for maintaining prospective memory, as they optimize the allocation of cognitive resources toward target-directed tasks [5]. 

However, the current results regarding ES deviate from prominent Australian and Dutch models. Specifically, a large-scale Australian study enrolling over 4,000 participants and a Dutch laboratory-based study of university students at Leiden University reported associations between ES and poorer cognitive outcome, including reduced verbal memory performance and diminished subjective recollection. These findings have been interpreted in the context of the cognitive demands involved in monitoring and regulating emotional expressions. [30, 31]

The discrepancy between the current findings and previous literature may be moderated by cultural and methodological factors. It has been highlighted that the efficacy and cognitive impact of ER strategies may vary across cultural contexts, as cultural norms regarding emotional expression may influence how suppression is experienced and used. In Egyptian and broader Arab collectivistic contexts, where emotional restraint may be more socially normative, ES may be experienced differently than in more individualistic, high-expressivity contexts such as Australia and the Netherlands [32]. 

Additionally, the divergence may arise from population characteristics. A Canadian study of 146 older adults with mild cognitive impairment (mean age 71) [33], a US 10-year prospective study of 468 middle-aged adults from the Midlife in the United States (MIDUS) cohort [34], and a US study examining physiological markers of ER in adults over 50 at the University of Rochester [8], all targeted pathological or age-related cognitive decline, examining factors such as mild cognitive impairment, perceived stress, and physiological markers. By contrast, the current study’s young, healthy academic population likely reflects metacognitive awareness rather than neurocognitive deficits, underscoring that the ER-SMC relationship is sensitive to both cohort age and cultural context.

From an educational and public health perspective, these findings may have implications for university infrastructure. Specifically, university counseling services could consider incorporating these insights to shift from purely reactive frameworks to proactive student support and well-being initiatives. Given that active engagement in ER is associated with a lower burden of SMCs, student mental health programs may benefit from strengthening students’ overall regulatory capabilities. Incorporating structured emotion regulation training campus-wide represents a potentially valuable avenue for supporting students facing high perceived academic and cognitive demands, although intervention studies would be required to determine whether such programs affect subjective memory outcomes.

In summary, this study has several strengths. Despite its limitations, it included a large sample of undergraduate students from multiple faculties and employed well-established, validated instruments to assess ER and SMP. Furthermore, it contributes to the existing literature by providing a large-scale, culturally contextualized analysis of the association between ER and SMP within an Egyptian undergraduate population. By identifying how regional paradigms and academic pressures uniquely relate to these cognitive-emotional dynamics, this work establishes a baseline framework for future cross-cultural comparisons and public health interventions.

### Study limitations

This study has several limitations that should be considered when interpreting the findings. Although faculties were selected using simple random sampling, participants within the selected faculties were recruited using convenience sampling from a single university, which may have introduced selection bias and limits the generalizability of the findings to students at other universities and the broader Egyptian university population. The cross-sectional design precludes any inference regarding the direction or causality of the observed associations between ER and SMP. Both ER and SMP were assessed using self-reported questionnaires, making the findings susceptible to recall bias and social desirability bias. Furthermore, SMP was not corroborated by objective cognitive assessments. Although previous research has shown that factors such as anxiety, depression, perceived stress, sleep quality, and social media use may influence both ER and SMP, these variables were not assessed in the present study. Consequently, residual confounding cannot be excluded, and the observed associations should be interpreted with caution. Future research should employ longitudinal designs to clarify the temporal relationship between ER and SMP. Multi-center studies involving students from different Egyptian universities would improve the generalizability of the findings. Additionally, incorporating objective neuropsychological assessments alongside self-reported measures would provide a more comprehensive evaluation of memory performance and help determine whether the observed associations extend to objective cognitive functioning.

## Conclusion

This cross-sectional study investigated the association between ER strategies and SMP, assessed via the PRMQ, among 1,287 Egyptian undergraduate students. Both CR and ES were independently and significantly associated with PRMQ scores in a multivariable model adjusting for sex, academic faculty, and family income, with greater reported use of either strategy associated with fewer self-reported everyday memory difficulties. Contrary to our initial hypothesis, the independent associations of CR and ES did not differ significantly in magnitude, suggesting that both regulation strategies relate comparably to subjective memory in this population. Although statistically significant, these associations were weak, and the model accounted for only approximately 10% of the variance in PRMQ scores, indicating that ER is one of several factors associated with SMCs. These findings point to an association between ER and subjective memory that warrants further investigation using longitudinal or experimental designs, as the cross-sectional design precludes causal inference. From an educational and public health perspective, ER may represent a modifiable factor of interest for university support services; further research, including intervention studies, would be needed to determine whether ER-focused programs affect subjective memory outcomes in this population.

## Supplementary Information

Below is the link to the electronic supplementary material.

Supplementary Material 1

Supplementary Material 2

## Data Availability

Data are available upon request from the corresponding author.

## Abbreviations

CR: Cognitive Reappraisal
ER: Emotion Regulation
ERQ: Emotion Regulation Questionnaire
ES: Expressive Suppression
IQR: Inter Quartile Range
MIDUS: Midlife in the United States
MSO: Microsoft Office
MTIS: Management Technology and Information Systems
PRMQ: Prospective and Retrospective Memory Questionnaire
RAND: Random Number Generation
SMCs: Subjective Memory Complaints
SMP: Subjective Memory Performance
STEM: Science, Technology, Engineering, and Mathematics
UAE: United Arab Emirates
UK: United Kingdom
US: United States
