# Supplementary material for: Emotion regulation and its association with subjective memory performance among undergraduate students at port said university
Source: BMC Psychol. 2026 Oct 19;14:1507. doi: 10.1186/s40359-026-05679-9 (PMC13682088; doi:10.1186/s40359-026-05679-9)
Supplement: Supplementary file 1 [file 40359_2026_5679_MOESM1_ESM.docx]

**تنظيم الانفعال الانفعالات والأداء الذاتي للذاكرة والتفاعل بينهما لدى طلاب جامعة بورسعيد**

يُجرى هذا البحث بواسطة مجموعة من أعضاء هيئة التدريس والهيئة المعاونة والطلاب بكلية الطب، جامعة بورسعيد. تهدف هذه الدراسة إلى استكشاف التفاعل بين تنظيم الانفعالات والأداء الذاتي للذاكرة لدى الطلاب الجامعيين بجامعة بورسعيد. بمشاركتك في هذا الاستبيان، فإنك توافق على شروط الدراسة وتتعهد بتقديم إجابات دقيقة وصادقة. نؤكّد لك أن جميع البيانات التي سيتم جمعها ستظل سرّية تمامًا ولن تُستخدم إلا لأغراض أكاديمية فقط. إن مشاركتك طوعية، ويحق لك الانسحاب في أي وقت دون أي عواقب**.**

| **أ. البيانات الاجتماعية الديموغرافية** | | | | | | | | | |  |
| --- | --- | --- | --- | --- | --- | --- | --- | --- | --- | --- |
| 1. **العمر :**...... 2. **الجنس:** ☐ ذكر ☐أنثى 3. **الجنسية:** ☐ مصري ☐ غير مصري 4. **الكلية:** | | | | | | | | | |  |
| - كلية الهندسة - كلية الطب - كلية العلوم - كلية الادارة والتكنولوجيا ونظم المعلومات | - كلية العلاج الطبيعي - كلية التربية النوعية - كلية الحقوق | | | | | | | | |  |
| 5. **السنة الدراسية:** ☐ الأولى ☐ الثانية ☐الثالثة ☐الرابعة ☐ الخامسة 6. **التقدير الدراسي:** ☐ امتياز ☐ جيد جدًا ☐ جيد ☐ مقبول ☐ راسب 7. **هل أنت مدخن:** ☐ مدخن حالي ☐ مدخن سابق ☐ غير مدخن 8. **استهلاك الكافيين:**☐ قهوة ☐ شاي ☐ أخرى ☐ لا شيء 9. **محل الإقامة:** ☐ حضري  ☐ريفي 10. **دخل الأسرة:** | | | | | | | | | |  |
| - مديون - يكفي الاحتياجات الأساسية | | - يكفي الاحتياجات الأساسية والطوارئ - الادخار والاستثمار | | | | | | | |  |
| **ب. استبيان تنظيم العواطف** | | | | | | | | | |  |
| **لكل عبارة، يرجى الإجابة باستخدام المقياس التالي:  (1= غير موافق بشدة، 2 = غير موافق، 3 = غير موافق إلى حد ما، 4 = محايد، 5 = موافق إلى حد ما، 6 = موافق، 7 = موافق بشدة)** | | | **1** | **2** | **3** | **4** | **5** | **6** | **7** | |
| **1**. عندما أريد أن أشعر بمزيد من المشاعر الإيجابية (مثل الفرح أو التسلية)، أغيّر ما أفكر فيه. | | | ☐ | ☐ | ☐ | ☐ | ☐ | ☐ | ☐ | |
| **2**. أحتفظ بمشاعري لنفسي. | | | ☐ | ☐ | ☐ | ☐ | ☐ | ☐ | ☐ | |
| **3**. عندما أريد أن أشعر بمشاعر أقل سلبية (مثل الحزن أو الغضب)، أغير ما أفكر فيه. | | | ☐ | ☐ | ☐ | ☐ | ☐ | ☐ | ☐ | |
| **4**. عندما أشعر بمشاعر إيجابية، أحرص على عدم التعبير عنها. | | | ☐ | ☐ | ☐ | ☐ | ☐ | ☐ | ☐ | |
| **5**. عندما أواجه موقفًا عصيبًا، أجعل نفسي أفكر فيه بطريقة تساعدني على الهدوء. | | | ☐ | ☐ | ☐ | ☐ | ☐ | ☐ | ☐ | |
| **6**.أنا أتحكم بمشاعري من خلال عدم التعبير عنها. | | | ☐ | ☐ | ☐ | ☐ | ☐ | ☐ | ☐ | |
| **7**. عندما أريد أن أشعر بمزيد من المشاعر الإيجابية، أغيّر طريقة تفكيري في الموقف. | | | ☐ | ☐ | ☐ | ☐ | ☐ | ☐ | ☐ | |
| **8**. أسيطر على مشاعري من خلال تغيير طريقة تفكيري في الموقف الذي أنا فيه. | | | ☐ | ☐ | ☐ | ☐ | ☐ | ☐ | ☐ | |
| **9**. عندما أشعر بمشاعر سلبية، أحرص على عدم التعبير عنها. | | | ☐ | ☐ | ☐ | ☐ | ☐ | ☐ | ☐ | |
| **10**. عندما أريد أن أشعر بمشاعر أقل سلبية، أغيّر طريقة تفكيري في الموقف. | | | ☐ | ☐ | ☐ | ☐ | ☐ | ☐ | ☐ | |

| **ج. استبيان الذاكرة المستقبلية المتوقعة\الاستعادية** | | | | | |
| --- | --- | --- | --- | --- | --- |
| **لكل عبارة، يرجى الإجابة باستخدام المقياس التالي: (1= في كثير من الأحيان، 2 = غالبًا، 3 = في بعض الأحيان، 4 = نادرًا، 5 = أبدًا)** | **1** | **2** | **3** | **4** | **5** |
| **1**. هل قررت فعل شيء في دقائق قليلة ومن ثم نسيت فعله؟ | ☐ | ☐ | ☐ | ☐ | ☐ |
| **2**. هل تفشل في التعرف على مكان زرته قبل ذلك؟ | ☐ | ☐ | ☐ | ☐ | ☐ |
| **3**. هل تفشل في فعل شيءٍ ما كان من المفترض أن تفعله بعد بضع دقائق على الرغم من كونه أمامك، مثل تناول أقراص الدواء أو إطفاء الغلاية؟ | ☐ | ☐ | ☐ | ☐ | ☐ |
| **4**. هل نسيت شيئًا قيل لك قبل بضع دقائق؟ | ☐ | ☐ | ☐ | ☐ | ☐ |
| **5**. هل تنسى المواعيد في حالة إن لم يذكرك بها أحدهم أو يجري تذكيرك بها من خلال برامج تذكير مثل التقويم أو المفكرة؟ | ☐ | ☐ | ☐ | ☐ | ☐ |
| **6**. هل تفشل في التعرف على الشخصيات في برنامج مذاع على المذياع أو التلفاز والتي قد ظهرت في مشاهد سابقة؟ | ☐ | ☐ | ☐ | ☐ | ☐ |
| **7**. هل تنسى شراء الأشياء التي خططت لشرائها مثل بطاقة تهنئة للأعياد الفطر أو الأضحى أو بيوم الميلاد، حتى ولو رأيت المتجر؟ | ☐ | ☐ | ☐ | ☐ | ☐ |
| **8**. هل تفشل في تذكر الأشياء التي حدثت خلال الأيام القليلة الماضية؟ | ☐ | ☐ | ☐ | ☐ | ☐ |
| **9**. هل تكرر القصة ذاتها للشخص ذاته في مناسبات مختلفة؟ | ☐ | ☐ | ☐ | ☐ | ☐ |
| **10**. هل تنوي أخذ شيء معك, قبل مغادرة الغرفة, ولكن بعدها بدقائق تتركه على الرغم من كونه أمامك؟ | ☐ | ☐ | ☐ | ☐ | ☐ |
| **11**. هل تضيع الأشياء التي وضعتها لتوك، مثل المجلة أو النظارات؟ | ☐ | ☐ | ☐ | ☐ | ☐ |
| **12**. هل تفشل في ذكر شيء، أو إعطائه لزائر قد طلب منك مناولته؟ | ☐ | ☐ | ☐ | ☐ | ☐ |
| **13**. هل تنظر لأشياء دون أن تدرك أنك قد رأيتها لوتك منذ لحظات؟ | ☐ | ☐ | ☐ | ☐ | ☐ |
| **14**. في حالة محاولتك الاتصال بصديق أو قريب وكان بالخارج, هل تنسى إعادة المحاولة في وقت لاحق؟ | ☐ | ☐ | ☐ | ☐ | ☐ |
| **15**. هل تنسى ما شاهدته على التلفاز باليوم السابق؟ | ☐ | ☐ | ☐ | ☐ | ☐ |
| **16**. هل تنسى إخبار أحدهم شيئًا كنت تعزم ذكره منذ دقائق مضت؟ | ☐ | ☐ | ☐ | ☐ | ☐ |
| **شكرًا لمشاركتك** | | | | | |
