# Supplementary material for: Emotion regulation and its association with subjective memory performance among undergraduate students at port said university
Source: BMC Psychol. 2026 Oct 19;14:1507. doi: 10.1186/s40359-026-05679-9 (PMC13682088; doi:10.1186/s40359-026-05679-9)
Supplement: Supplementary file 2 [file 40359_2026_5679_MOESM2_ESM.docx]

| **Table 2: Median (Q1–Q3) scores of the Emotion Regulation Questionnaire (ERQ).** | | | | |
| --- | --- | --- | --- | --- |
|  | | | **Quartiles** | |
|  | **Possible score** | **Median** | **Q1** | **Q3** |
| **Cognitive reappraisal:** |  |  |  |  |
| 1. When I want to feel more positive emotion (such as joy or amusement), I change what I’m thinking about. |  | 4 | 2.00 | 6.00 |
| 3. When I want to feel less negative emotion (such as sadness or anger), I change what I’m thinking about. |  | 5 | 4.00 | 6.00 |
| 5. When I’m faced with a stressful situation, I make myself think about it in a way that helps me stay calm. |  | 5 | 3.00 | 6.00 |
| 7. When I want to feel more positive emotion, I change the way I’m thinking about the situation. |  | 5 | 3.00 | 6.00 |
| 8. I control my emotions by changing the way I think about the situation  I’m in. |  | 5 | 3.00 | 6.00 |
| 10. When I want to feel less negative emotion, I change the way I’m thinking about the situation. |  | 5 | 3.50 | 6.00 |
| **Expressive suppression:** |  |  |  |  |
| 2. I keep my emotions to myself. |  | 5 | 4.00 | 7.00 |
| 4. When I am feeling positive emotions, I am careful not to express them. |  | 3 | 1.00 | 5.00 |
| 6. I control my emotions by not expressing them. |  | 4 | 2.00 | 6.00 |
| 9. When I am feeling negative emotions, I make sure not to express them. |  | 5 | 3.00 | 6.00 |
| **Total:** |  |  |  |  |
| Total score: | 10-70 | 44 | 37 | 51 |
| Total CR score: | 6-42 | 27 | 22 | 32 |
| Total ES score: | 4-28 | 17 | 13 | 21 |
| Q1: First Quartile  Q3: Third Quartile | | | | |

| **Table 3: Median (Q1–Q3) scores of the Prospective-Retrospective Memory Questionnaire (PRMQ).** | | | | | | |
| --- | --- | --- | --- | --- | --- | --- |
|  | | | | | **Quartiles** | |
|  | | | **Possible score** | **Median** | **Q1** | **Q3** |
| **Items:** | | |  |  |  |  |
| 1. Do you decide to do something in a few minutes’ time and then forget to do it? | | |  | 4 | 1.00 | 5.00 |
| 2. Do you fail to recognise a place you have visited before? | | |  | 3 | 2.00 | 4.00 |
| 3. Do you fail to do something you were supposed to do a few minutes later even though it’s there in front of you, like take a pill or turn off the kettle? | | |  | 3 | 2.00 | 4.00 |
| 4. Do you forget something that you were told a few minutes before? | | |  | 3 | 1.00 | 4.00 |
| 5. Do you forget appointments if you are not prompted by someone else or by a reminder such as a calendar or diary? | | |  | 3 | 2.00 | 4.00 |
| 6. Do you fail to recognise a character in a radio or television show from scene to scene? | | |  | 3 | 2.00 | 4.00 |
| 7. Do you forget to buy something you planned to buy, like a birthday card, even when you see the shop? | | |  | 3 | 2.00 | 4.00 |
| 8. Do you fail to recall things that have happened to you in the last few days? | | |  | 3 | 2.00 | 4.00 |
| 9. Do you repeat the same story to the same person on different occasions? | | |  | 3 | 2.00 | 4.00 |
| 10. Do you intend to take something with you, before leaving a room or going out, but minutes later leave it behind, even though it’s there in front of you? | | |  | 3 | 1.00 | 4.00 |
| 11. Do you mislay something that you have just put down, like a magazine or glasses? | | |  | 3 | 2.00 | 4.00 |
| 12. Do you fail to mention or give something to a visitor that you were asked to pass on? | | |  | 3 | 2.00 | 4.00 |
| 13. Do you look at something without realising you have seen it moments before? | | |  | 3 | 2.00 | 4.00 |
| 14. If you tried to contact a friend or relative who was out, would you forget to try again later? | | |  | 3 | 2.00 | 4.00 |
| 15. Do you forget what you watched on television the previous day? | | |  | 3 | 2.00 | 4.00 |
| 16. Do you forget to tell someone something you had meant to mention a few minutes ago? | | |  | 3 | 1.00 | 4.00 |
| **Subscales:** | | |  |  |  |  |
| Prospective | Short-term | Self | 2-10 | 7 | 6.00 | 9.00 |
|  |  | Environment | 2-10 | 6 | 5.00 | 8.00 |
|  | Long-term | Self | 2-10 | 6 | 5.00 | 8.00 |
|  |  | Environment | 2-10 | 6 | 5.00 | 8.00 |
| Retrospective | Short-term | Self | 2-10 | 7 | 5.00 | 8.00 |
|  |  | Environment | 2-10 | 6 | 5.00 | 7.00 |
|  | Long-term | Self | 2-10 | 6 | 4.00 | 8.00 |
|  |  | Environment | 2-10 | 6 | 4.00 | 7.00 |
| **Total:** | | |  |  |  |  |
| Total Prospective score | | | 8-40 | 26 | 21 | 30 |
| Total Retrospective score | | | 8-40 | 24 | 20 | 29 |
| Total score | | | 16-80 | 50 | 43 | 58.50 |
| Q1: First Quartile  Q3: Third Quartile | | | | | | |
